# Effect of Peer Education on Early Breast Cancer Detection, Health Responsibility, Health Beliefs, Knowledge, and Practices Among University Students

**DOI:** 10.1111/phn.70000

**Published:** 2025-06-16

**Authors:** Sabahat Coskun, Nisa Alibekiroğlu, Gamzenur Gençyürek

**Affiliations:** ^1^ Faculty of Health Sciences Şeyh Edebali University Bilecik Turkey; ^2^ Faculty of Health Sciences Nursing Department Şeyh Edebali University Bilecik Turkey

**Keywords:** breast cancer, early diagnosis, health belief, health responsibility, peer education, self‐examination

## Abstract

**Background:**

Breast cancer remains a major global health issue, with early detection playing a key role in reducing mortality rates. University students represent an important population for promoting health responsibility and preventive behaviors such as breast self‐examination (BSE). Peer education has emerged as a promising strategy to enhance health‐related knowledge and practices among young adults.

**Objective:**

This study aims to evaluate the impact of peer education on early breast cancer detection, health responsibility, health beliefs, knowledge, and practices among university students.

**Methods:**

This randomized controlled experimental study involved 244 students (124 in the intervention group and 120 in the control group). Data were collected using a Demographic Information Form, the Comprehensive Breast Cancer Knowledge Test (CBCKT), the Champion's Health Belief Model (CHBM), and a BSE skill assessment test. The intervention group received education on breast cancer and BSE. Both groups were assessed 6 months after the intervention. Data analysis included frequency, percentage, mean, standard deviation, Chi‐square, eta squared, independent samples *t*‐test, and paired samples *t*‐test.

**Results:**

Before the peer education, no significant differences were observed between the groups. However, following the intervention, significant improvements were noted in breast cancer knowledge, health responsibility, health beliefs, and BSE practices in the intervention group. Additionally, while only 4.8% of students performed BSE in 12 steps before the intervention, this increased to 58.1% after the education.

**Conclusions:**

Peer education significantly enhanced breast cancer knowledge, health responsibility, health beliefs, and BSE practices. Nurses play a vital role in supporting these interventions, promoting early detection, and improving health outcomes through targeted education strategies.

## 1 Introduction

Breast cancer is the most commonly diagnosed cancer among women both globally and in Turkey (WHO 2022). Despite the high incidence of breast cancer in developed countries, mortality rates remain comparatively low. This difference is largely due to the advanced capabilities for early detection, screening, and treatment available in these countries (Bray et al. 2018). Early detection and awareness significantly enhance treatment success rates and make a substantial contribution to patients' quality of life. The most common screening methods for early detection of breast cancer include breast self‐examination (BSE), clinical breast examination (CBE), and mammography (Özdemir and Ünal 2023). Since breast masses are often detected by individuals themselves, performing BSE is important (Kifle et al. 2016). BSE is a simple, low‐cost, and safe method that helps women familiarize themselves with their bodies and detect unusual changes early (İncesu et al. 2024). When performed regularly, BSE can detect up to 40% of breast lesions (Kifle et al. 2016). However, the number of women who regularly perform BSE remains low (Tahmasebi and Noroozi 2016; Özdemir and Ünal 2023). This is due to factors such as a lack of knowledge on how to perform the method, insufficient awareness of breast cancer symptoms and risk factors, and a lack of supportive factors for regular BSE (Aydın et al. 2021).

Encouraging BSE among all women from a young age and ensuring its regular practice is considered a crucial nursing responsibility for the early detection of cancer (Mahadevaiah et al. 2021). It is believed that this responsibility should be instilled in nurses starting from their student years. BSE is one of the essential nursing skills to be taught to nursing students. These professional skills are developed not only in clinical settings but also through practical field experiences. Nursing students, who represent the future, play a crucial role in developing health behaviors that contribute to public health and in spreading their knowledge. Educational institutions offer ideal environments for providing training on BSE, given their ability to reach large audiences.

One of the most effective models in health education and promotion is the Health Belief Model (HBM). The HBM is a key approach used to understand and alter individuals' health behaviors. It highlights the role of perceived threats and benefits in influencing behavior change (Champion 1999; Gözüm and Çapık 2014). This study aims to enhance the knowledge and practices of young women at the university regarding breast cancer and BSE through peer education programs delivered by nursing students, based on the HBM. Peer education is recognized as an important concept in the nursing profession (Cust and Guest 2020).

Peer education, grounded in social learning theory, aims to enhance young people's interactions with their peers and foster better self‐awareness. This approach focuses on training volunteer youth on specific topics and encouraging them to share their knowledge and experiences (Ayran et al. 2017). Peer education is a highly effective method for promoting health behaviors learned from peers. Open communication among young people creates a safe learning environment, even for topics considered taboo or private (Ceylan and Koç 2021). This approach can help young individuals gain knowledge about BSE and foster the development of health responsibility behaviors. A study in this field has demonstrated that peer education improves knowledge and practices related to BSE (Ayran et al. 2017).

Participation in peer education programs from the early stages of nursing education not only enhances students' professional knowledge and skills but also enables them to make more significant contributions to public health after graduation. The knowledge and awareness acquired during this process empower nurses to deliver more effective services to the community throughout their professional careers.

This study aims to investigate the impact of peer education‐based programs on young individuals' early detection of breast cancer, health responsibility, and health behaviors. The findings are expected to provide valuable contributions to the field of public health. Additionally, the study seeks to contribute to the nursing and health education literature, offer a fresh perspective to the existing body of knowledge, and generate positive effects on women's health and, more broadly, on public health.

### 1.1 Aim and Hypotheses

This study aims to investigate the effectiveness of peer education in enhancing university students’ knowledge, beliefs, and practices regarding early breast cancer detection, as well as their health responsibility and self‐examination skills.

The hypotheses of the study are as follows:

H 1a Students in the intervention group will have significantly higher scores on the Comprehensive Breast Cancer Knowledge Test (CBCKT) than those in the control group.

H 1b : Students in the intervention group will have significantly higher scores on the health responsibility subscale than those in the control group.

H 2a : Students in the intervention group will score significantly higher on the perceived susceptibility subscale of the Champion's Health Belief Model (CHBM) compared to the control group.

H 2b : Students in the intervention group will score significantly higher on the perceived severity subscale compared to the control group.

H 2c : Students in the intervention group will score significantly higher on the perceived benefits subscale compared to the control group.

H 2d : Students in the intervention group will score significantly higher on the perceived confidence/self‐efficacy subscale compared to the control group.

H 2e : Students in the intervention group will score significantly higher on the health motivation subscale compared to the control group.

H 2f : Students in the intervention group will score significantly lower on the perceived barriers subscale compared to the control group.

H 3 : Students in the intervention group will demonstrate significantly improved BSE skills compared to their pre‐test performance.

## 2 Methods

### 2.1 Design

This randomized controlled trial was conducted among university students. The independent variable is the “peer education model,” while the dependent variables include scores from the CBCKT, Health Responsibility Scale, CHBM subscales, and BSE practice skills.

### 2.2 Study Population and Sample Size

The study population consisted of students enrolled at a public university (*N* = 280). To ensure sufficient statistical power, the sample size was calculated using the G*Power 3.1 Power Analysis Program. The primary outcome measure was the perceived benefit subscale score of the HBM Scale for Breast Cancer and BSE among university students. Data from previous studies (Kayar 2019) provided mean scores and standard deviations for the benefit subscale before (14.48 ± 4.24) and after (16.07 ± 0.96) the intervention, which were used to calculate the effect size. Based on these parameters—Type I error rate (α) = 0.05, statistical power (1‐β) = 0.95, and effect size (d) = 0.51—the minimum required sample size was determined to be 99 participants per group, totaling 198 participants. However, a total of 280 students (140 in the intervention group and 140 in the control group) who met the inclusion criteria and provided informed consent were initially recruited. This was done to account for possible attrition, incomplete responses, and voluntary withdrawal. Although the literature generally recommends an oversampling rate of 10%–20% (Suresh and Chandrashekara 2012), a higher rate was deliberately adopted in this study to preserve internal validity and ensure sufficient statistical power, particularly given the follow‐up nature and behavioral focus of the educational intervention.

#### 2.2.1 Inclusion and Exclusion Criteria

The inclusion criteria for the study included young women who (1) had no history of any cancer, including breast cancer, (2) had no communication barriers, (3) were not pregnant or currently breastfeeding, and (4) voluntarily agreed to participate in the study. The exclusion criteria were as follows: (1) individuals with a prior diagnosis of any type of cancer, (2) participants who had previously received education on breast cancer and BSE, and (3) participants in the intervention group who attended fewer than two sessions.

### 2.3 Randomization and Group Allocation

**FIGURE 1:**
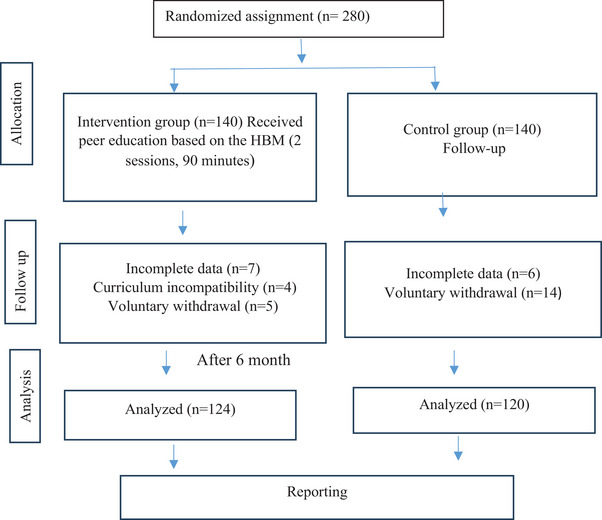
Flow of the participants in the study. HBM = Health Belief Model. [Colour figure can be viewed at wileyonlinelibrary.com]

This study was designed as an open‐label, randomized controlled trial. A total of 280 female students who met the inclusion criteria participated in the study at a state university. The university consists of six faculties and two vocational schools, excluding the Faculty of Health Sciences and the Faculty of Medicine. To minimize the risk of contamination, students were recruited from different faculties. However, both the intervention and control groups consisted exclusively of students enrolled in four‐year undergraduate programs to ensure educational equivalence across groups. To ensure allocation concealment, the sealed envelope method was employed. During the randomization process, the names of all included faculties were placed in sealed envelopes and randomly selected by an independent researcher. The first selected faculty was assigned to the intervention group, while the second selected faculty was assigned to the control group. This method was implemented to ensure unbiased group allocation. Additionally, the randomization sequence was generated by an independent researcher to maintain impartiality in the allocation process. Out of the 280 initially enrolled students, 244 (intervention: *n* = 124, control: *n* = 120) completed the study. Thirty‐six students (attrition rate: 12.86%) withdrew during the final assessment phase due to curriculum incompatibility, voluntary withdrawal, or missing data. The intervention group received peer education, whereas no intervention was applied to the control group. The health beliefs, health responsibility, knowledge levels, and practices of both groups were assessed before and after the intervention using standardized and validated scales (Figure 1). Due to the nature of the intervention, blinding was not feasible.

### 2.4 Data Collection Tools

#### 2.4.1 Information Form

The questionnaire, developed by the researchers based on a literature review (Yurt et al. 2019; Kayar 2019), consists of two sections. The first section contains 14 questions related to socio‐demographic characteristics and factors influencing breast cancer and BSE (e.g., family history of breast cancer, personal practice of BSE). The second section includes 12 questions focused on knowledge and practices concerning the signs of breast cancer and BSE.

#### 2.4.2 Health‐Promoting Lifestyle Profile II (HPLP II)/Health Responsibility Subscale

The HPLP II, initially developed by Walker et al. in 1987 and revised in 1996, was adapted into Turkish by Bahar et al. in 2008. The scale consists of 52 items across six subscales: spiritual growth, health responsibility, physical activity, nutrition, interpersonal relationships, and stress management, using a 4‐point Likert scale. In this study, only the “Health Responsibility” subscale was utilized, with scores ranging from 9 to 36. Higher scores indicate greater health responsibility (Bahar et al. 2008). The Health Responsibility Subscale showed high internal consistency (*α* = 0.843) in this study.

#### 2.4.3 CBCKT

CBCKT, developed by Stager in 1993, has a Cronbach's alpha coefficient of 0.71. The Turkish version's validity and reliability were established by Başak in 2015, with a Cronbach's alpha of 0.90. The scale comprises two subscales: general knowledge and treatability of breast cancer. The first 12 questions assess general knowledge about breast cancer, while questions 13–20 focus on its treatability. The scale uses a true/false format, awarding 1 point for correct answers and 0 points for incorrect or unanswered questions. Higher scores indicate greater knowledge about breast cancer (Başak 2015). Although the Cronbach's alpha for the CBCKT was moderate (*α* = 0.577) in this study, the scale's multidimensional content likely contributed to this result. This is a known limitation of comprehensive knowledge tests, as internal consistency tends to decrease when items assess diverse constructs within a single tool (Boateng et al. 2018).

#### 2.4.4 Champion Health Belief Model Scale (CHBMS)

CHBMS, developed by Victoria Champion in 1984 and revised in 1993, 1997, and 1999, is designed to assess women's beliefs and attitudes towards breast cancer and BSE. The Turkish adaptation was performed by Karayurt and Dramalı in 2007. The scale consists of 42 items across six subscales, using a 5‐point Likert scale: Perceived Susceptibility (3 items): Assesses perceived individual risk of breast cancer. Perceived Severity (7 items): Measures the perceived seriousness of breast cancer. Perceived Benefits (4 items): Evaluates perceived advantages of performing BSE. Perceived Barriers (11 items): Identifies perceived obstacles to performing BSE. Self‐Efficacy (10 items): Assesses perceived competence in detecting abnormal breast masses through BSE. Health Motivation (7 items): Reflects the individual's concern and interest in their health. Each subscale is evaluated separately, and no total score is calculated. Lower scores in the Perceived Barriers subscale, alongside higher scores in the other subscales, indicate positive attitudes and beliefs about breast cancer and BSE. The Cronbach's alpha reliability coefficients for the Turkish version range from 0.58 to 0.89 across subscales (Champion 1999). In this study, the internal consistency of the CHBMS subscales was found to be acceptable to high, with Cronbach's alpha coefficients as follows: perceived susceptibility (*α* = 0.793), perceived severity (*α* = 0.840), perceived benefits (*α* = 0.839), perceived barriers (*α* = 0.740), confidence/self‐efficacy (*α* = 0.847), and health motivation (*α* = 0.752).

#### 2.4.5 BSE Knowledge Questionnaire

A knowledge questionnaire was developed by the researchers for the post‐test phase, based on a literature review (Ayran et al. 2017; General Directorate of Public Health 2024), to evaluate participants' skills in performing BSE. The questionnaire consists of 12 items that assess the steps involved in BSE. One point is awarded for each correct answer, and zero points for incorrect answers. This form was administered to the experimental group before and after the educational intervention. The total score can range from 0 to 12. In this study, the internal consistency of the questionnaire was found to be high, with a Cronbach's alpha coefficient of 0.978.

### 2.5 Procedure and Intervention

This study employed the “Near Peer” model for selecting peer educators. The selection criteria included academic excellence, successful completion of the Public Health Nursing course, and voluntary participation. Based on these criteria, two peer educators were chosen, and a specialized training program was developed to prepare them for the intervention. The training incorporated informational booklets, case presentations, and visual materials to enhance learning. The program comprised four 45‐min sessions covering breast cancer risk factors, early detection methods, and the steps of BSE). Interactive teaching strategies were employed to strengthen the educators’ knowledge and skills. Prior to the intervention, pre‐test assessments were conducted online in an auditorium designated by a faculty member from the sampled faculties. Participants in both the intervention and control groups completed a Knowledge Form, the Health Responsibility Subscale, the CBCKT, the CHBMS, and the BSE Knowledge Questionnaire to evaluate their knowledge, attitudes, and behaviors. These assessments established a baseline for measuring changes following the intervention. Initially, the training was designed to be delivered in four 45‐min sessions. However, due to logistical constraints and students’ curriculum schedules, the experimental group was divided into two subgroups, with each receiving two 45‐min face‐to‐face training sessions. Due to scheduling constraints, the initially planned four‐session intervention was delivered as two extended sessions, with content and objectives fully preserved. The sessions featured a range of educational tools, including PowerPoint presentations, verbal lectures, video screenings, and interactive Q&A discussions. The training content encompassed female reproductive system anatomy, breast cancer risk factors, and the significance of early detection. A comprehensive overview of BSE steps was provided, along with discussions on both established and emerging risk factors. To ensure clarity, additional explanations on risk factors and symptoms were given, and real‐life case studies were integrated to reinforce theoretical knowledge through practical application. The control group did not receive any training or intervention during this period. Six months post‐intervention, follow‐up assessments were conducted by distributing post‐test forms online to both groups. Data collection occurred between October 2023 and June 2024. Upon completion of the study, and in accordance with ethical research principles, the entire training content (including PowerPoint presentations, educational videos, and additional learning materials used in face‐to‐face sessions) was made available to the control group via a secure, web‐based platform. This ensured that all participants received equal access to the educational benefits of the intervention, while maintaining the integrity of the study design by preventing cross‐contamination during data collection.

### 2.6 Ethical Considerations

The study was approved by the Non‐Interventional Research Ethics Committee of Bilecik Şeyh Edebali University (Date: 29/06/2020; Ethical Approval No: 54674167‐050.01.04; Decision No: 10 from Meeting No: 8). In addition, institutional permission was obtained prior to the commencement of the study. Written and verbal informed consent was obtained from all participants, ensuring their voluntary participation. The confidentiality of all collected data was meticulously safeguarded, and participants' rights‐including the right to withdraw from the study at any time‐were fully respected. The research was conducted in full compliance with the ethical principles of the Declaration of Helsinki, maintaining the highest ethical standards throughout the study.

### 2.7 Data Analysis

Data analysis was conducted using SPSS version 25.0 software. Descriptive statistics, including frequencies, percentages, mean ± standard deviation, and minimum‐maximum values, were utilized to summarize the data. The normality of the distribution was assessed by examining the skewness and kurtosis values. Data were considered normally distributed if the skewness and kurtosis values were within the range of −2 to +2 (George and Mallery 2019). For data exhibiting normal distribution, independent *t*‐tests were employed to assess differences between independent groups. Paired *t*‐tests were used to examine within‐group differences for dependent groups. To analyze categorical variables, Pearson Chi‐square tests were applied, as they are appropriate for determining the significance of group distributions in categorical data. Additionally, the effect size was calculated to determine the magnitude of differences between group means, using eta squared (*η* ^2^). Eta squared quantifies the proportion of variance in the dependent variable that is explained by the independent variable, with values ranging from 0 to 1. Effect size interpretation followed these guidelines: *η* ^2^ values of 0.01 indicate a small effect, 0.06 a moderate effect, and 0.14 or higher denote a large effect. In this study, *η* ^2^ values reached up to 0.225, demonstrating a mix of small, moderate, and large effects between group means. Statistical significance was set at *p* < 0.05, ensuring that the likelihood of the results being due to chance was less than 5%, confirming the statistical significance of the findings. Furthermore, all assumptions underlying the statistical tests were verified, and the dataset was deemed appropriate for analysis. A per‐protocol analysis approach was employed in this study. Only participants who completed both the pre‐test and post‐test assessments and fully participated in the intervention were included in the final analysis. Although 280 students were initially recruited, data from 124 students in the intervention group and 120 in the control group were analyzed due to voluntary withdrawals, incomplete participation, and missing data. The intention‐to‐treat (ITT) method was not applied, as the objective of this study was to evaluate the efficacy of the intervention under full adherence conditions. Per‐protocol analysis is commonly preferred in behavioral and educational studies for this purpose (Gupta 2011; Abraha and Montedori 2010). The overall rate of missing data remained below 10% and is unlikely to have introduced significant bias.

## 3 Results

### 3.1 Findings on the Descriptive Characteristics of the Students

A total of 280 students were initially enrolled in the study. However, 36 students withdrew due to curriculum incompatibility, incomplete data, or voluntary withdrawal. Consequently, the final analysis included 244 participants, with 124 in the experimental group and 120 in the control group (Figure 1). There were no significant differences between the experimental and control groups in terms of income, alcohol consumption, family history of breast cancer, or frequency of BSE practice (*p* > 0.05). BSE awareness was higher in the control group (68.3% vs. 57.3%, *p* = 0.074), but this difference was not statistically significant. The most common reason for not practicing BSE was lack of knowledge on how to perform it (experimental group: 83.9%, control group: 71.7%), with a significant difference between the groups (*p* = 0.094). Regarding BSE information sources, the experimental group reported a higher proportion of individuals who had not received any BSE information (59.7% vs. 43.3%). The control group relied more on mass media for BSE information (36.7% vs. 24.7%), while other sources like healthcare professionals and family/friends were less common in both groups. Overall, the findings indicate that BSE awareness and practice were generally low in both groups (Table 1). There were no significant differences between the experimental and control groups regarding the reasons for performing BSE, the age to begin BSE, or the timing of BSE for postmenopausal and premenopausal women (*p* > 0.05). Similarly, awareness of breast cancer symptoms, including breast hardness, skin changes, nipple redness or thickening, inward retraction, changes in nipple position, and lumps in the armpit, remained at similar levels across both groups (*p* > 0.05) (Table 2).

**TABLE 1:** Comparison of socio‐demographic characteristics between the experimental and control groups (*n* = 244).

Socio‐demographic characteristics	Experimental group (*n* = 124)	Control group (*n* = 120)	*χ* ^2^	*p*
**Income status**				
Low income	15 (12.1%)	14 (11.7%)	0.11	0.917
Middle income	109 (87.9%)	106 (88.3%)		
**Alcohol consumption**				
Yes	16 (12.9%)	11 (9.2%)	0.865	0.352
No	08 (87.1%)	109 (90.8%)		
**Tobacco consumption**				
Yes	29 (23.4%)	17 (14.2%)	3.389	0.066
No	95 (76.6%)	103 (85.9%)		
**Family history of breast cancer**				
Yes	19 (15.3%)	10 (8.3%)	2.845	0.092
No	105 (84.7%)	110 (91.7%)		
**Awareness of BSE**				
Yes	71 (57.3%)	82 (68.3%)	3.199	0.074
No	53 (42.7%)	38 (31.7%)		
**BSE information source**				
No source	74 (59.7)	52 (43.3)	6.993	0.072
Mass media	30 (24.7)	44 (36.7)		
Health professionals	13 (10.5)	14 (11.7)		
Family and friends	7 (5.6)	10 (8.3)		
**Frequency of BSE practice**				
Never	108 (87.1%)	96 (80.0%)	4.375	0.122
Once a month	6 (4.8%)	4 (3.3%)		
Once every 3 months	10 (8.1%)	20 (16.7%)		
**Reasons for not practicing BSE**				
Don't know how to perform it	104 (83.9%)	86 (71.7%)	6.392	0.094
No perceived issues	4 (3.2%)	6 (5.0%)		
Fear	15 (12.1%)	23 (19.2%)		
Forgetfulness	1 (0.8%)	5 (4.2%)		
**Knowledge of clinical breast exam**				
Aware	3 (2.4%)	2 (1.7%)	0.172	0.678
Unaware	121 (97.6%)	118 (98.3%)		
**Knowledge of mammography**				
Aware	14 (11.3%)	16 (13.3%)	0.236	0.627
Unaware	110 (88.7%)	104 (86.7%)		

Abbreviations: BSE = breast self‐examination, *χ*2 = Chi square.

**TABLE 2:** Comparison of BSE knowledge and practices and breast cancer symptoms between the experimental and control groups before the intervention (*n* = 244).

BSE knowledge area	Experimental group (*n* = 124) *n* (%)	Control group (*n* = 120) *n* (%)	*χ* ^2^	*p*
**Reason for performing BSE**				
Aware	107 (%86.3)	109 (%90.8)	1.239	0.266
Unaware	17 (%13.7)	11 (%9.2)		
**Age to begin BSE**				
Aware	21 (%16.9)	22 (%18.3)	0.082	**0.774**
Unaware	103 (%83.1)	98 (%81.7)		
**Time for postmenopausal women to perform BSE**				
Aware	3 (%2.4)	5 (%4.2)	0.587	0.444
Unaware	121 (%97.6)	115 (%95.8)		
**Time for premenopausal women to perform BSE**				
Aware	6 (%4.8)	4 (%3.3)	0.352	0.555
Unaware	118 (%95.2)	116 (%96.7)		
**Regular BSE practice**				
Yes	6 (%4.8)	3 (%2.5)	0.939	0.333
No	118 (%95.2)	117 (%97.5)		

**Breast cancer symptom knowledge**	**Experimental group (*n* = 124)** ***n* (%)**	**Control group (*n* = 120)** ***n* (%)**	***χ* ^2^**	***p***
**Hardness or lump in breast for >2 weeks**				
Aware	71 (%57.3)	77 (%64.2)	1.220	0.269
Unaware	53 (%42.7)	43 (%35.8)		
**Skin changes: thickening, swelling, or color**				
Aware	64 (%51.6)	63 (%52.5)	0.019	0.890
Unaware	60 (%48.4)	57 (%47.5)		
**Nipple redness, ulceration, or thickening**				
Aware	44 (%35.5)	34 (%28.3)	1.434	0.231
Unaware	80 (%64.5)	86 (%71.7)		
**Inward retraction of breast/nipple**				
Aware	27 (%21.8)	31 (%25.8)	0.555	0.456
Unaware	97 (%78.2)	89 (%74.2)		
**Nipple position changes**				
Aware	27 (%21.8)	27 (%22.5)	0.019	0.891
Unaware	97 (%78.2)	93 (%77.5)		
**Lumps in armpit**				
Aware	61 (%49.2)	67 (%55.8)	1.078	0.299
Unaware	63 (%50.8)	53 (%44.2)		
**Discharge from nipple (brown, black, blood)**				
Aware	40 (%32.3)	41 (%34.2)	0.100	0.752
Unaware	84 (%67.7)	79 (%65.8)		

Abbreviations: BSE = breast self‐examination, *χ*2 = Chi square.

### 3.2 Findings Related to the Hypotheses of the Research

Table 3 presents a comparison of BSE knowledge and awareness of breast cancer symptoms between the experimental and control groups following the intervention. In the experimental group, awareness regarding the reasons for performing BSE significantly increased, showing a meaningful difference compared to the control group (*p* = 0.047). Knowledge about the appropriate age to begin BSE, as well as the timing for postmenopausal and premenopausal women to perform BSE, was significantly higher in the experimental group, with these differences being statistically significant (*p* < 0.001). Furthermore, the rate of regular BSE practice was notably higher in the experimental group compared to the control group (*p* < 0.001). In terms of breast cancer symptoms, awareness increased significantly after the intervention. The experimental group demonstrated higher levels of awareness regarding symptoms such as breast hardness or lumps, skin changes, nipple redness or thickening, inward retraction, changes in nipple position, lumps in the armpit, and discharge from the nipple (brown, black, or blood) compared to the control group, with all differences being statistically significant (*p* < 0.05). These results suggest that the intervention effectively enhanced both BSE knowledge and awareness of breast cancer symptoms.

**TABLE 3:** Comparison of BSE knowledge and practices regarding breast cancer symptoms between the experimental and control groups after the intervention (*n* = 244).

BSE knowledge area	Experimental group (*n* = 124) *n* (%)	Control group (*n* = 120) *n* (%)	*χ* ^2^	*p*
**Reason for performing BSE**				
Aware	122 (%98.4)	112 (%93.3)	3.963	0.047
Unaware	2 (%1.6)	8 (%6.7)		
**Age to begin BSE**				
Aware	78 (%62.9)	21 (%17.5)	52.139	**0.001**
Unaware	46 (%37.1)	99 (%82.5)		
**Time for postmenopausal women to perform BSE**				
Aware	55 (%44.4)	8 (%6.7)	45.226	0.001
Unaware	69 (%55.6)	112 (%93.3)		
**Time for premenopausal women to perform BSE**				
Aware	71 (%57.3)	7 (%5.8)	74.154	**0.001**
Unaware	53 (%42.7)	113 (%94.2)		
**Regular BSE practice**				
Yes	72 (%58.1)	3 (%2.5)	88.438	0.001
No	52 (%41.9)	117 (%97.5)		

**Breast cancer symptom knowledge**	**Experimental group (*n* = 124)** ***n* (%)**	**Control group (*n* = 120)** ***n* (%)**	***χ* ^2^**	***p***
**Hardness or lump in breast for >2 weeks**				
Aware	97 (%78.2)	79 (%65.8)	4.659	**0.031**
Unaware	27 (%21.8)	41 (%34.2)		
**Skin changes: thickening, swelling, or color**				
Aware	105 (%84.7)	67 (%55.8)	24.392	0.001
Unaware	19 (%15.3)	53 (%39.0)		
**Nipple redness, ulceration, or thickening**				
Aware	96 (%77.4)	40 (%33.3)	48.043	0.001
Unaware	28 (%22.6)	80 (%66.7)		
**Inward retraction of breast/nipple**				
Aware	77 (%62.1)	32 (%26.7)	30.973	0.001
Unaware	47 (%37.9)	88(%73.3)		
**Nipple position changes**				
Aware	71 (%57.3)	31 (%25.8)	24.754	0.001
Unaware	53 (%42.7)	89 (%74.2)		
**Lumps in armpit**				
Aware	86 (%69.4)	59 (%49.2)	10.308	0.001
Unaware	38 (%30.6)	61 (%50.8)		
**Discharge from nipple (brown, black, blood)**				
Aware	73 (%58.9)	48 (%40.0)	8.687	0.003
Unaware	51 (%41.1)	72 (%60.0)		

Abbreviations: BSE = breast self‐examination, *χ*2 = Chi square.

Table 4 presents a comparison of pretest and posttest scores for the CBCKT, health responsibility, and health beliefs between the experimental and control groups. Prior to the intervention, there were no significant differences between the groups in terms of CBCKT scores, health responsibility, sensitivity, seriousness perception, perceived benefits, perceived barriers, self‐efficacy, and health motivation (*p* > 0.05). However, following the intervention, significant improvements were observed in the experimental group compared to the control group. Specifically, the experimental group demonstrated significantly higher posttest scores in the following areas: CBCKT (14.03 ± 2.23 vs. 13.30 ± 2.07, *p* = 0.009, *ƞ* ^2^ = 0.028), health responsibility (19.87 ± 4.49 vs. 18.54 ± 3.90, *p* = 0.014, *ƞ* ^2^ = 0.025), sensitivity (8.37 ± 1.40 vs. 7.70 ± 1.95, *p* = 0.002, *ƞ* ^2^ = 0.038), seriousness perception (23.20 ± 4.26 vs. 21.42 ± 4.37, p = 0.002, *ƞ* ^2^ = 0.041), perceived benefits (17.76 ± 1.61 vs. 16.82 ± 2.01, *p* = 0.001, *ƞ* ^2^ = 0.060), self‐efficacy (35.36 ± 5.34 vs. 29.85 ± 4.90, *p* = 0.001, *ƞ* ^2^ = 0.225), and health motivation (27.07 ± 3.25 vs. 24.75 ± 4.02, *p* = 0.001, *ƞ* ^2^ = 0.092). These results suggest that the intervention had a significant positive impact on participants' breast cancer knowledge, health responsibility, and health beliefs.

**TABLE 4:** Comparison of pretest and posttest scores for CBCKT, health responsibility, and health beliefs between experimental and control groups (*n* = 244).

Variable	Experimental group (X̄ ± SD)	Control group (X̄ ± SD)	*t*	*p*	Eta kare (*ƞ*2)	95% CI	
						Lower	Upper
**Pre‐test**							
CBCKT	12.63 ± 2.93	13.21 ± 2.06	1.777	0.077	N/A	−0.06	1.22
Health responsibility	18.47 ± 5.00	18.79 ± 4.37	0.524	0.601	N/A	−0.86	1.50
Sensitivity	7.55 ± 1.87	7.61 ± 2.00	0.243	0.808	N/A	−0.42	0.54
Seriousness perception	20.85 ± 5.64	21.00 ± 4.80	0.216	0.829	N/A	−1.17	1.46
Perceived benefit	16.63 ± 2.79	16.38 ± 2.43	−0.755	0.451	N/A	−1.91	0.40
Perceived barrier	27.62 ± 5.29	26.91 ± 4.60	−1.107	0.270	N/A	−1.95	0.54
Self−efficacy	29.75 ± 6.33	29.65 ± 5.50	−0.142	0.887	N/A	−1.60	1.39
Health motivation	24.60 ± 4.06	25.15 ± 3.91	1.066	0.288	N/A	−0.46	1.55
**Post‐test**							
CBCKT	14.03 ± 2.23	13.30 ± 2.07	−2.652	0.009	0.028	−1.27	−0.18
Health responsibility	19.87 ± 4.49	18.54 ± 3.90	−2.485	0.014	0.025	−2.40	−0.27
Sensitivity	8.37 ± 1.40	7.70 ± 1.95	−3.110	0.002	0.038	−1.10	−0.24
Seriousness perception	23.20 ± 4.26	21.42 ± 4.37	−3.211	0.002	0.041	−2.86	−0.68
Perceived benefit	17.76 ± 1.61	16.82 ± 2.01	−3.915	0.001	0.060	−1.41	−0.46
Perceived barrier	25.80 ± 5.63	26.78 ± 4.48	1.496	0.136	N/A	−0.30	−2.25
Self‐efficacy	35.36 ± 5.34	29.85 ± 4.90	−8.389	0.001	0.225	−6.80	−4.21
Health motivation	27.07 ± 3.25	24.75 ± 4.02	−4.965	0.001	0.092	−3.24	−1.40

*Note*: Eta kare (ƞ2 d is provided only for significant variables).

Abbreviations: BSE = breast self‐examination, CBCKT = comprehensive breast cancer knowledge test, CHBM = Champion's Health Belief Model, CI = confidence Interval for the mean difference between groups, N/A = not applicable (no significant result), *t* = Independent sample *t*‐test.

The results from Table 5 show a significant improvement in BSE skill performance in the experimental group (*n* = 124) following the training. Prior to the training, most participants exhibited low performance on BSE steps, with percentages ranging from 4.8% to 12.9%. After the training, there was a notable increase in the execution of each step, with percentages ranging from 39.5% to 58.1%. All improvements were statistically significant (*p* < 0.001), highlighting the effectiveness of the training program in enhancing participants' ability to correctly perform BSE. These findings demonstrate a clear benefit in improving both BSE awareness and skill acquisition.

**TABLE 5:** Comparison of BSE skill performance steps before and after training in the experimental group (*n* = 124).

BSE skill performance steps	Pre‐traning *n* (%)	Post‐traning *n* (%)	*t*	*p*
1. Stand in front of a mirror in a well‐lit room with the upper body exposed. (BSE is recommended 5−7 days after the start of menstruation).	14 (11.3%)	72 (58.1%)	−10.397	.001
2. Stand in front of the mirror with arms relaxed at the sides. Inspect both breasts and nipples for symmetry, color changes, retraction, or peau d'orange.	12 (9.7%)	69 (55.6%)	−9.624	0.001
3. Stand in front of the mirror with arms raised. Check both breasts and nipples for symmetry and any indentation.	10 (8.1%)	70 (56.5%)	−10.738	0.001
4. Stand in front of the mirror with hands on hips, applying light pressure to the lower back. Assess the size, symmetry, and retraction of the breasts and nipples.	11 (8.9%)	68 (54.8%)	−9.913	0.001
5. Stand in front of the mirror, lean slightly forward, and assess the size, symmetry, and any retraction of the breasts and nipples.	13 (10.5%)	69 (55.6%)	−10.064	0.001
6. During the breast examination, the pads of the index, middle, and ring fingers are used.	13 (10.5%)	65 (52.4%)	−9.425	0.001
7. During the examination, the fingers are kept together and remain in constant contact with the skin.	15 (12.1%)	64 (51.6%)	−8.964	0.001
8. While standing for BSE, place the hand of the examined side on the head.	16 (12.9%)	70 (56.5%)	−9.741	0.001
9. Using the index, middle, and ring fingers, begin at the nipple and perform circular motions with varying pressure to palpate the breast from the clavicle to the breastfold and from the axilla to the sternum.	11 (8.9%)	58 (46.8%)	−8.382	0.001
10. Gently squeeze the nipples to check for any discharge (brown, black, bloody, or clear) and press the nipple to assess for firmness.	8 (6.5%)	67 (54.0%)	−10.566	0.001
11. Place the hand of the examined side on the waist and use the index, middle, and ring fingers of the other hand to palpate the axillary lymph nodes.	10 (8.1%)	61 (49.2%)	−9.270	0.001
12. While lying on the back, place a small pillow under the shoulder of the examined side and repeat the breast examination	6 (4.8%)	49 (39.5%)	−8.081	0.001

Abbreviations: BSE = breast self‐examination, *t* = paired samples *t*‐test.

Based on these findings:

- H1a and H1b were accepted, indicating significant improvements in CBCKT and health responsibility.
- H2a to H2e were accepted, confirming that the intervention effectively enhanced students' perceived susceptibility, severity, benefits, self‐efficacy, and health motivation.
- H2f was rejected, as the difference in perceived barriers was not statistically significant.
- H3 was accepted, supporting that BSE skills significantly improved post‐intervention.

These hypothesis‐based outcomes were used to structure the Discussion section accordingly, providing a more organized interpretation of how each component of the intervention influenced specific outcomes and aligning the findings with existing literature.

## 4 Discussion

Breast cancer is the most common cancer among women, and early detection is crucial for effective treatment and improved survival outcomes (Aydın et al. 2021). However, evidence suggests that breast cancer in young women progresses more aggressively, often leading to poorer prognoses (Ranganath et al. 2023). Given this context, the present study examines the role of peer education in improving university students' awareness of breast cancer, BSE skills, health responsibility, and health beliefs.

In this study, peer education was employed as an intervention to enhance knowledge about breast cancer and promote BSE skills among university students. Initially, no significant differences were observed between the experimental and control groups. However, following the peer education intervention, the experimental group exhibited significant improvements in health responsibility, health beliefs, and BSE knowledge and practices. These findings corroborate the work of Ericson, who emphasized the pivotal role of peer influence in shaping identity and personal values during late adolescence. Peer education has been identified as a particularly effective approach for fostering mutual support and self‐confidence (Korkut and Başer 2020). The positive outcomes of this study align with similar research (Ayran et al. 2017; El Fttah Ali and Hussein 2018; Yurt et al. 2019), reinforcing the effectiveness of the peer education model.

Before the intervention, the majority of students demonstrated limited knowledge regarding breast cancer risk factors and BSE. Additionally, BSE practices were found to be suboptimal, likely due to a lack of understanding about how and when to perform BSE, as well as fear and limited awareness issues consistently highlighted in the literature (Ibitoye and Thupayegale‐Tshwenegae 2021; Khiyali et al. 2017; Nde et al. 2015). After the peer education intervention, there was a marked improvement in both BSE knowledge and practices, demonstrating the intervention's effectiveness in bridging these knowledge gaps. Peer education fosters an environment that encourages information sharing, providing a supportive setting that enhances learning and behavior change. The findings suggest that peer education not only boosts BSE knowledge and practices but also promotes health responsibility behaviors. By encouraging regular and correct BSE, peer education empowers students to take an active role in their health. The importance of BSE in early breast cancer detection is underscored by existing literature, which indicates that most breast cancer cases are self‐reported by individuals (Sadoh et al. 2021). The significant improvements observed in the CBCKT and in health responsibility scores among students in the intervention group provide empirical support for Hypotheses H1a and H1b, confirming the effectiveness of the peer education approach in enhancing both knowledge and health‐related responsibility.

An analysis of information sources revealed that most students relied on mass media to gain knowledge about BSE. This aligns with Nde et al. (2015), who emphasized the role of mass media in educating women about the significance and proper technique of BSE. Furthermore, 10.5% of participants in the experimental group and 11.7% in the control group reported receiving information about BSE from healthcare professionals. These findings highlight the critical need for healthcare professionals to take a more proactive role in promoting BSE practices, particularly in light of the effectiveness of peer education in disseminating health information.

HBM offers a valuable framework for understanding how beliefs, values, and attitudes shape health behaviors, including participation in early detection screenings and adherence to preventive health measures (Gözüm and Çapık 2014). In this study, after the peer education intervention, the experimental group showed significant increases in perceived susceptibility and severity of breast cancer compared to the control group (Table 4). These results align with findings from Khiyali et al. (2017), who found that higher levels of perceived susceptibility and severity increase the likelihood of engaging in protective health behaviors. However, Kıssal and Kartal (2019) found no significant changes in perceived susceptibility or severity, which may be attributed to young women's lower perception of breast cancer risk compared to older women. Additionally, the experimental group reported significant increases in perceived benefits, supporting previous research (Khiyali et al. 2017; Akhtari‐Zavare et al. 2016). However, some studies (Kıssal and Kartal 2019) have reported no significant changes in perceived benefits, suggesting that prior knowledge levels and post‐intervention support may influence outcomes.

Regarding perceived self‐efficacy, which reflects individuals' confidence in their ability to perform BSE correctly and effectively, a significant improvement was observed in the experimental group. This finding is consistent with previous studies (Akhtari‐Zavare et al. 2016; Khiyali et al. 2017). In contrast, Kıssal and Kartal (2019) did not observe a significant difference in self‐efficacy, which they attributed to a lack of practice opportunities. In our study, peer education enhanced students' confidence in performing BSE, likely due to increased awareness of its importance.

Health motivation, defined as the general willingness and intention to improve one's health, was significantly higher in the experimental group following the intervention. This increase further underscores the effectiveness of peer education in fostering health motivation. Although previous literature presents varying findings on this topic (Akhtari‐Zavare et al. 2016; Kayar 2019; Kıssal and Kartal 2019), the observed difference in this study can be attributed to the peer education model, which encouraged students to share information, engage in discussions, and support one another in adopting preventive health behaviors.

Regarding perceived barriers, although there was a decrease in scores in the experimental group compared to the control group after the intervention, this difference was not statistically significant (Table 4). This finding is consistent with Kıssal and Kartal (2019), while other studies (Khiyali et al. 2017; Akhtari‐Zavare et al. 2016) have reported significant reductions in perceived barriers following educational interventions. Future research should further explore these barriers and adapt educational programs to address them more effectively. Overall, the significant increases observed in perceived susceptibility, severity, benefits, self‐efficacy, and health motivation (H2a–H2e) demonstrate the successful application of the HBM within the context of this intervention. In contrast, Hypothesis H2f was not supported, as the change in perceived barriers was not statistically significant following the peer education program.

BSE skills were assessed before and after the intervention (Table 5). Prior to the peer education intervention, the BSE implementation rate was 4.8%, but it increased to 58.1% following the intervention. Although the majority of students followed the correct steps, not all performed the examination with complete accuracy. Nonetheless, there was a significant improvement in BSE skills, with students demonstrating greater consistency in performing BSE, identifying abnormalities, and assessing breast tissue correctly (Table 5). BSE skill performance significantly improved in the intervention group, with correct step execution increasing markedly after training. This finding supports Hypothesis H3, confirming that structured peer education can enhance students' ability to apply BSE techniques accurately and confidently. These results align with similar studies (Ayran et al. 2017; Azuonwu and Uka‐Nnodim 2022; Ibitoye and Thupayegale‐Tshwenegae 2021), which also observed significant increases in BSE rates following educational interventions. These findings highlight the potential of peer education as an effective strategy to raise awareness about breast cancer and encourage the widespread adoption of BSE.

In summary, the study's findings support the majority of the proposed hypotheses and confirm that peer education is a valuable and practical method for enhancing breast cancer awareness, health responsibility, and self‐care skills among university students.

### 4.1 Limitations

One limitation of this study is that it was conducted with female students from a single university, which may limit the generalizability of the findings to a wider population of young adult females. Another limitation is the reliance on self‐reported data, which may introduce biases in behavior reporting. Additionally, longer follow‐up periods (e.g., 3, 6, and 12 months) are needed to assess the long‐term effects and sustainability of the intervention. However, due to the transition to remote education after the earthquake, the follow‐up period was limited to 6 months.

## 5 Conclusion

This study provides evidence that peer education conducted by trained nursing students is an effective intervention to improve university students’ knowledge, beliefs, and skills regarding breast cancer and BSE. The intervention significantly increased health responsibility, enhanced perceived susceptibility, severity, benefits, and motivation, and improved BSE performance.

These findings suggest that public health nursing programs can integrate short‐term, structured peer education models into university settings to promote early detection of behaviors among young women. Public health nurses should supervise and guide peer‐led initiatives, ensuring content accuracy and continuity. In practice, offering regular peer training sessions on BSE within academic schedules and using brief but focused sessions can make these interventions both scalable and cost‐effective.

Future implementations should focus on reducing perceived barriers to BSE and expanding the program through digital platforms and mHealth tools to increase accessibility. More practice‐oriented training and ongoing reinforcement could further strengthen self‐efficacy and long‐term adoption of preventive behaviors.

## Author Contributions

S.C.: Study design. S.C., N.A., and G.G.: Data collection, development of educational content. N.A. and G.G.: Study implementation. S.C.: Data analysis, study supervision, drafting of manuscript, and critical revisions for Important Intellectual Content.

## Data Availability

The data that support the findings of this study are available on request from the corresponding author. The data are not publicly available due to privacy or ethical restrictions.
